# Cataract Surgery in a Case of Bilateral Idiopathic Corneal Ascher Rings Associated With Anterior Segment Changes

**DOI:** 10.1155/crop/8164915

**Published:** 2026-04-02

**Authors:** Anca Delia Pantalon

**Affiliations:** ^1^ Ophthalmology Clinic, St. Spiridon Emergency University Hospital, Iasi, Romania; ^2^ Pediatric Ophthalmology and Strabismus Department, Moorfields Eye Hospital NHS Foundation Trust, London, UK, nhs.uk

## Abstract

**Aim:**

The aim of this study is to report an extremely rare case of corneal annular opacities, incidentally found in a patient referred for age related cataract surgery. Additional anterior segment particularities were detected in this patient.

**Methods:**

Clinical and imaging methods were employed for a rigorous ophthalmological examination; a full systemic check‐up has been included in the investigation panel, as well.

**Results:**

Examinations revealed in a 69‐year‐old patient bilateral and symmetrical corneal ring opacities, within the full stromal thickness, with gray‐whitish appearance and ellipsoid shape; diameters were 4–6 mm, clear central/peripheral cornea; no other corneal structural changes were identified via anterior segment optical coherence tomography (AS‐OCT). Specular microscopy parameters, including pachymetry, were unremarkable. Such corneal changes could not be classified as sequelae of infectious keratitis, corneal dystrophies, or degenerations. Systemic pathologies have been also excluded; existent high blood pressure (HBP) and hypercholesterolemia could not be linked to such corneal findings in our patient. By exclusion, the corneal opacities were classified as Ascher rings. Cataract surgery was uneventful, with excellent recovery. IOL power calculation reflected the axial anysometropia (21.5D in OD, respectively, 15D in OS–Clareon SY60WF, Alcon), as well as asymmetric gonioscopy findings.

**Conclusion:**

Cataract surgery performed in the presence of Ascher corneal rings alone, does not pose additional risks during phacoemulsification or postoperative intervals. However, association with anterior segment changes (narrow anterior chamber angle, axial anysometropia) might raise additional surgical concerns, though unrelated to the rare corneal findings. These associations have not been reported in any previous cases from literature.

## 1. Introduction

Corneal opacities are often found in the context of various pathologies: ocular or systemic (degenerations, dystrophies, infections, immune intracorneal reactions, drug‐induced, and systemic metabolic disorders), postsurgical healing processes (e.g., refractive corneal procedures) or as posttraumatic anterior segment changes (e.g., corneal lacerations) [1]. We report hereby an extremely rare type of corneal opacity, which is diagnosed by exclusion.

Ascher rings have been described initially in 1964 [2], but there are less than 20 cases mentioned in the literature, at the moment [3]. There is no genetic transmission [4], they are typically bilateral and stable in time [3] with negative laboratory investigations and incidental discovery.

## 2. Case Report

A 69‐year‐old female patient was referred to our clinic in November 2023 for age related cataract surgery. Written informed consent was obtained from the patient for publication of this case report and any accompanying images.

Despite a decrease in her vision starting 2–3 years before the current examination, our patient claimed previous excellent driving skills with full vision in both eyes. Any other ocular symptoms have been denied as well.

Slit lamp biomicroscopy, fundus examination, intraocular pressure (IOP) by Goldmann tonometry, and optic biometry (Zeiss IOL Master 700; Carl Zeiss AG, Oberkochen, Germany) were routinely performed.

Anterior segment examination revealed bilateral corneal opacities (Figure 1a,b), placed within the full thickness corneal stroma, with ellipsoid shape (longer axis at 8–02 o′clock meridian) and gray‐whitish appearance; diameter was 4/6 mm in both eyes; central/peripheral cornea were clear with unaltered sensitivity. There were no signs of anterior chamber (AC) flare or cellularity and no evidence of thinning, neovascularization, or corneal scarring. An asymmetry of the AC depth was visible (occludable angle [OD] < OS) by initial Van Herick assessment.

Figure 1Preoperatory OD (a), OS (b) lens status and Ascher corneal ring (partially visible).

**Figure:**
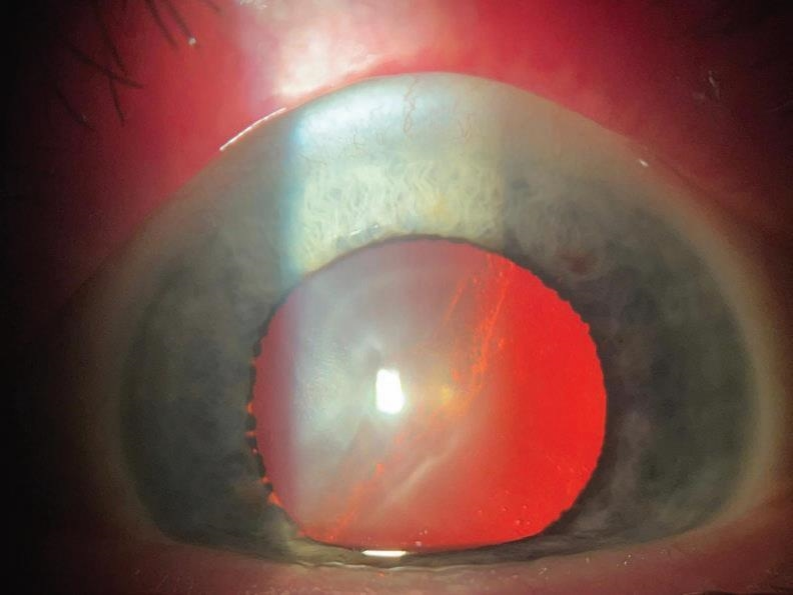
(a)

**Figure:**
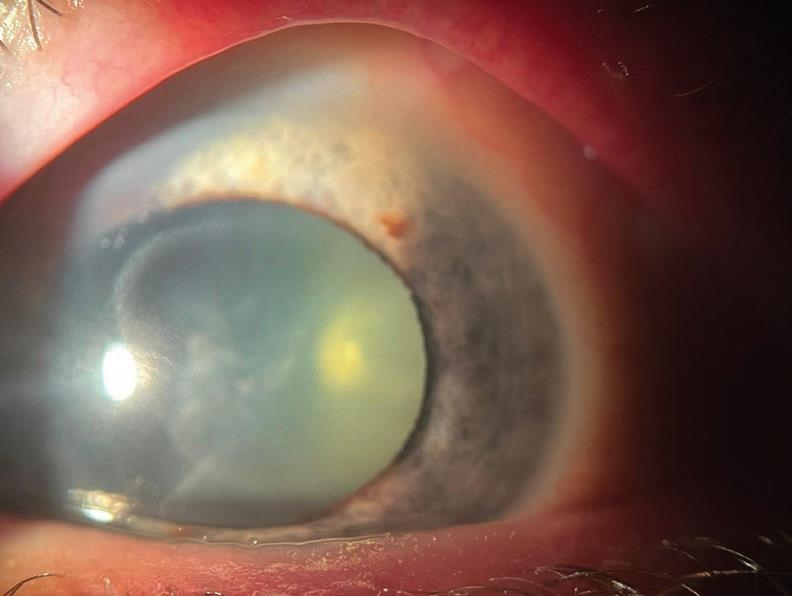
(b)

Imaging methods: Pentacam (Oculus Inc., Wetzlar, Germany) and Spectral Domain Anterior Segment OCT (SD AS‐OCT, REVO‐NX, Optopol) identified the corneal opacity characteristics, but revealed no other structural changes.

Ophthalmological examination with all baseline parameters is summarized in Table 1.

**Table 1:** Baseline parameters.

Parameter	OD	OS
BCVA (decimal)	0.5	0.3
Refraction	sph +3 ≈ cyl +0.75/170^a^	sph +1.25 ≈ cyl −1.50/120^a^
Keratometry (D)	43.50	43.50
AL (mm)	23.48	25.51
ACD (mm)	2.39	3.20
L (mm)	4.99	3.92
IOP (mmHg)	17	14
IOL power (D)	21.5	15
Pachy (*μ*m)	556	555
ECC (cells/mm^2^)	2722	2754

Abbreviations: ACD, anterior chamber depth; AL‐axial lengths; BCVA, best corrected visual acuity; D, dioptry; ECC, endothelial cells count/mm^2^ (specular microscopy); IOL, intraocular lens; IOP‐intraocular pressure; L‐lens thickness; OD‐right eye (lat. oculus dexter), OS‐left eye (lat. oculus sinister), Pachy‐Pachymetry (specular microscopy).

^a^Degrees for cylinder.

Bilateral keratometry and pachymetry by Pentacam assessment were within normal limits (available as supplemental material), as well as unremarkable specular microscopy (Nidek, CM 530) parameters (CD > 2700 cells/mm^2^), with comparable coefficient of variation (CV < 32*%*) and similar hexagonality (> 70%), available as supporting information (Figure S1 and S2).

Corneal tomograms acquired by SD AS‐OCT illustrate in Figure 2a,b full thickness stromal opacities, corresponding to the Ascher rings areas, with normal epithelium and endothelium. Characteristics of the corneal changes could not be classified as sequelae of infectious keratitis, corneal dystrophies or degenerations after ocular examinations and the patient′s history.

Figure 2Corneal imaging OD (a), OS (b) by SD AS‐OCT REVO‐NX, Optopol; highly reflective material present in full depth corneal stroma (yellow arrows); no epithelial/endothelial involvement.

**Figure:**
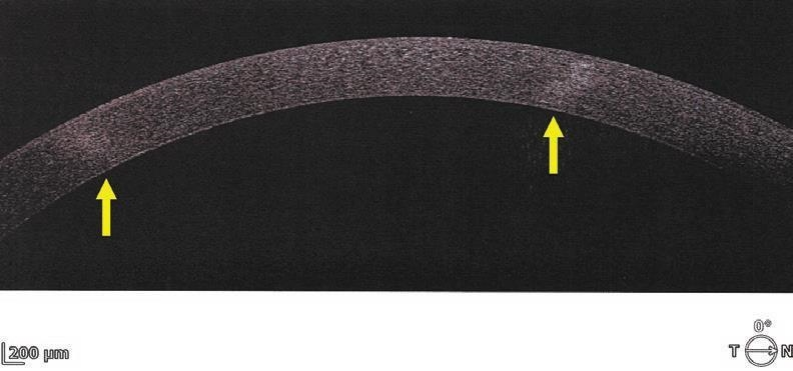
(a)

**Figure:**
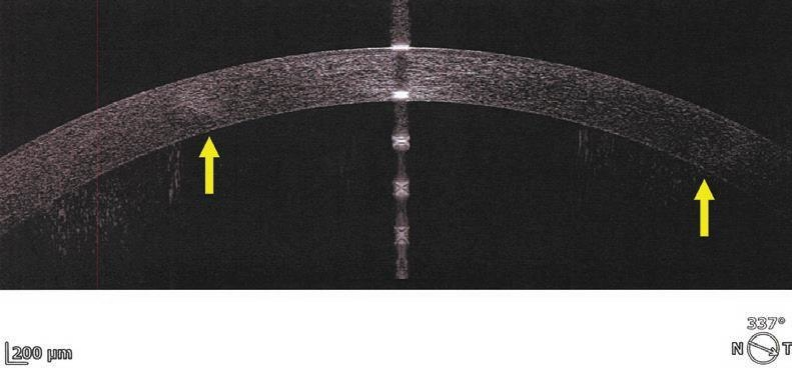
(b)

Fundus examination revealed pink, healthy optic discs in both eyes with retinal vascular changes typical for Stage II hypertensive retinopathy, but otherwise unremarkable. C/D ratio was 0.2 in both eyes.

Subjectively, the preoperative gonioscopy revealed a narrower angle in OD versus OS by lens anterior vaulting mechanism, objectively quantified by Pentacam measurements: anterior chamber angle (ACA) was 18° versus 29° in OD versus OS. Lens thickness (LT) measured via optical biometry showed a difference of > 1 mm between eyes; see Table 1 for exact data. IOL power calculation reflected the axial anysometropia identified in our patient (21.5D in OD and 15D in OS—Clareon SY60WF, Alcon).

Cataract surgery with monofocal IOL implantation was sequentially performed (Figure 3a,b), first in OD, then in OS with excellent bilateral outcome (BCVA = 1). Her IOPs remained within normal limits pre and postoperatively and there were no changes compatible with glaucoma, or any signs of previous inflammation in the anterior segment that might point out towards intermittent angle closures in any of the eyes. Pentacam imaging and ACA remeasurements proved a wide opening of the angle after cataract extraction in OD (Figure 3c,d).

Figure 3(a) OD—postoperative aspect (PC‐IOL, bag), Ascher ring fully visible upon mid‐dilated pupil, (b) OS—preoperative status, Ascher ring fully visible upon pupil dilation, (c) Pentacam imaging OD—postoperative wide ACA opening; Ascher rings visible (yellow arrows), (d) Pentacam imaging OS—preoperative status; Ascher rings visible (yellow arrows).

**Figure:**
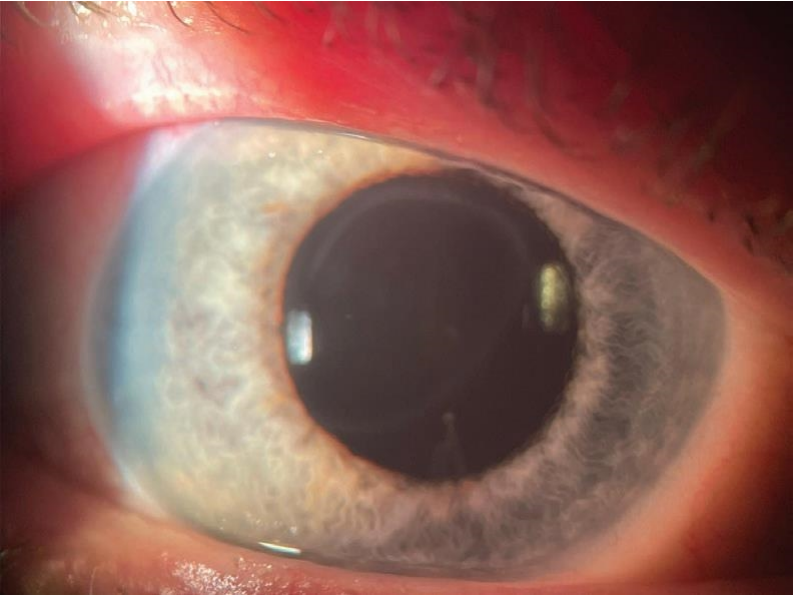
(a)

**Figure:**
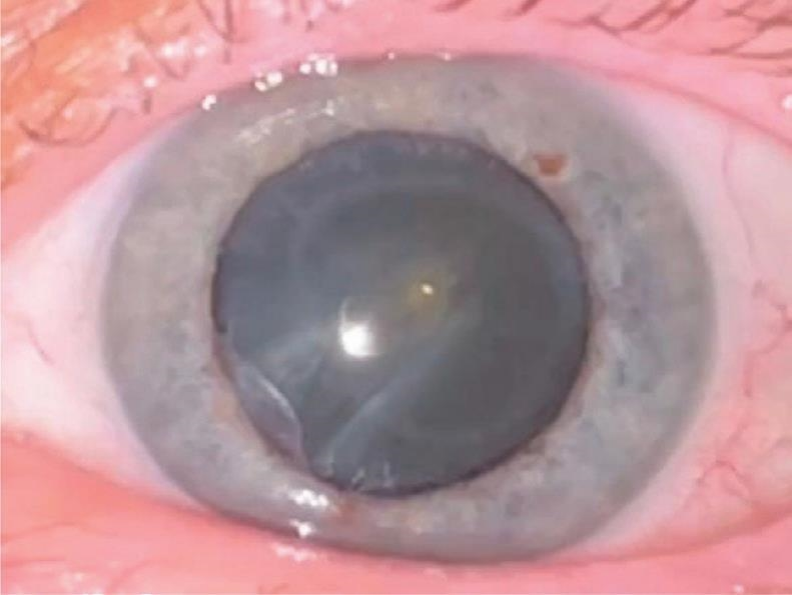
(b)

**Figure:**
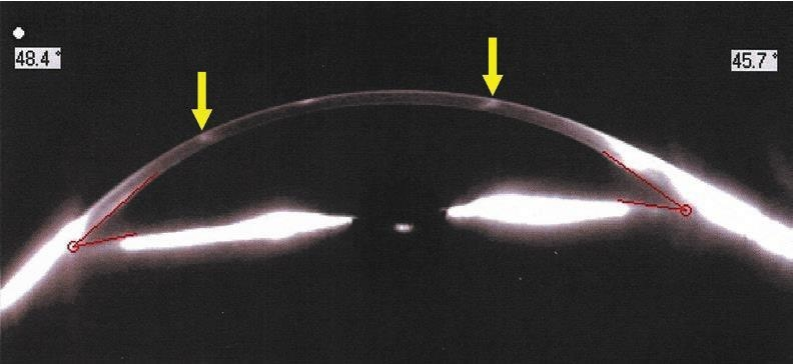
(c)

**Figure:**
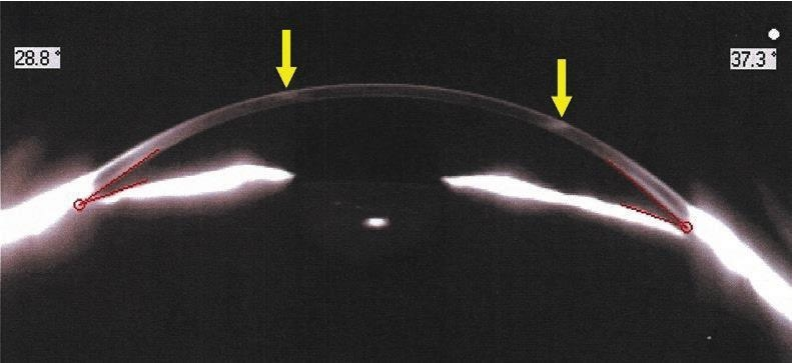
(d)

An extensive systemic checkup has been performed for our patient. Her high blood pressure and hypercholesterolemia were well controlled with medication (beta‐blockers, sartans and statins). All other clinical exams/laboratory tests (blood, urine, biochemistry, autoimmune/metabolic markers, etc.) were inconclusive for any systemic pathology.

## 3. Discussion

Stromal corneal opacities offer ample place for discussion and differential diagnosis. Ascher corneal rings are extremely rare entities [3], incidentally found in adult eyes [4]. Proven stable in time, they offer excellent functional prognosis for the patients where/when present [3]. Given the appearance and the topography, the diagnosis is only by exclusion after a detailed medical history and thorough examinations with extensive laboratory work‐up ruling out corneal inflammations, degenerations/dystrophies, or systemic diseases.

Shape is circular or oval [5, 6], involving various depths of the stroma: anterior [2], posterior [7], and full thickness [6–8], with a white‐grayish appearance and clear demarcation from the surrounding corneal tissue [9]. Variability has been cited among Ascher rings, as one author identified multiple (four), bilateral opacities [9], whereas other authors discuss about single and unilateral changes [10].

The ring size is variable (4–10 mm) [5], its width reaching about 500 *μ* [11] with normal cornea inside and outside the rings [4]. Sagittal corneal tomograms (AS OCT) found a homogenous structure/reflectivity with triangular shape [11], apex pointing towards the epithelium and the base towards the endothelium [4]. Yet, some authors found granular changes (variable reflectivity) within the rings [12]. Confocal microscopy research identified nonprogressive, abnormal extra/intracellular microdeposits of unknown etiology in the corneal tissue corresponding to the rings; proposed theory hypothesized about errors in collagen fibrils formation; hence no connection with inflammatory or infiltrative pathologies [12]. Other imaging methods (e.g., ultrasound biomicrosopy) described comparable acoustic reflectivity between the Ascher ring zone and the neighboring normal tissue [12].

No systemic findings are typically associated with these corneal changes [13]; however, hypercholesterolemia has been described in some cases [4, 14, 15], including ours.

Our case associated ocular changes in the anterior segment (asymmetric narrow angles and axial anysometropia) that changed the typical approach for the order of cataract surgery in our patient, starting with the narrower, OD. However, the sequential phacoemulsification procedure was 2 weeks apart and offered the patient full visual recovery; the presence of the Ascher rings did not change the surgical plan/approach. Other reported cases cite ocular associations for the posterior segment, for example, glial tufts in the optic disc [16], peripheral retinal hole [16], choroidal nevus [17], or unspecified refractive errors [3].

To the best of our knowledge this is the first case report to mention the association between Ascher rings with axial anysometropia and gonioscopic asymmetric changes.

## 4. Conclusion

Cataract surgery considered in the presence of Ascher corneal rings alone does not pose additional risks during phacoemulsification or postoperative intervals. However, association with anterior segment changes (narrow angle, axial anysometropia) might raise additional surgical concerns, but is unrelated to the rare corneal incidental findings. These associations have not been reported in the previous cases found in literature.

## Funding

No funding was received for this manuscript.

## Ethics Statement

Ethics Committee approval was waived for this case report, in accordance with local institutional Ethics Committee and national guidelines (standard of practice in our hospital).

ConsentWritten informed consent was obtained from the patient for publication of the details of their medical case and any accompanying images.

## Conflicts of Interest

The authors declare no conflicts of interest.

## Supporting Information

Additional supporting information can be found online in the Supporting Information section.

## Supporting information


**Supporting Information 1** Figure S1: Corneal topography, keratometry and pachymetry data by Pentacam imaging — normal values in both eyes.

**Supporting Information 2** Figure S2: Specular microscopy data (Nidek, CM 530®)— normal range in both eyes.

## Data Availability

Research data are not publicly available on legal or ethical grounds. Further inquiries regarding clinical, imaging, and laboratory data can be directed to the corresponding author; data are available upon request.

## References

[1] American Academy of Ophthalmology , External Disease and Cornea (Section 8), in Basic and Clinical Science Course, 2017, American Academy of Ophthalmology, San Francisco.

[2] Ascher K. W. , An Unusual Corneal Ring, Ber Zusammenkunft Dtsch Ophthalmol Ges. (1964) 65, 44–46, 14260575. 14260575

[3] Megalla M. , Li E. , Branden P. , and Chow J. , Bilateral Idiopathic Corneal Opacity: A Report of Ascher Ring and a Review of the Literature, American Journal of Ophthalmology Case Reports. (2021) 23, 101176, 10.1016/j.ajoc.2021.101176, 34368499. 34368499 PMC8326342

[4] Melles G. R. , de Sera J. P. , Eggink C. A. , Cruysberg J. R. M. , and Binder P. S. , Bilateral, Anterior Stromal Ring Opacity of the Cornea, British journal of ophthalmology. (1998) 82, no. 5, 522–525, 10.1136/bjo.82.5.522, 2-s2.0-0031802480, 9713059. 9713059 PMC1722592

[5] Nofal N. , Darvish-Zargar M. , and Teboul B. , A Case of Symmetric Bilateral Ring-Shaped Corneal Opacities, Canadian Journal of Ophthalmology. (2022) 57, no. 2, e42–e44, 10.1016/j.jcjo.2021.07.006, 34419420. 34419420

[6] Bron A. J. , Peripheral Ring Opacity of the Cornea, British Journal of Ophthalmology. (1969) 53, no. 4, 270–273, 10.1136/bjo.53.4.270, 2-s2.0-0014494815, 5781036. 5781036 PMC1207311

[7] Bopp S. and Laqua H. , Corneal Ascher Ring. A Ring-Shaped Stromal Corneal Opacity, Klinische Monatsblatter fur Augenheilkunde. (1991) 198, no. 03, 201–204, 10.1055/s-2008-1045952, 2056739. 2056739

[8] Moshirfar M. , Corneal Surgical Problem, Journal of Cataract and Refractive Surgery. (2015) 41, no. 4, 895–896, discussion 899 10.1016/j.jcrs.2015.03.003, 2-s2.0-84926314090. 25840313

[9] Caroline P. J. and Melles G. R. , Two Cases of Bilateral, Stromal Ring Opacity of the Cornea, Cornea. (2001) 20, no. 2, 237–238, 10.1097/00003226-200103000-00029, 2-s2.0-0035113657, 11248840. 11248840

[10] Uddaraju M. , Mascarenhas J. , das M. , and Prajna N. V. , A Case of Bilateral, Multiple, Symmetric, Concentric Ring-Shaped Opacities in the Cornea, JAMA Ophthalmol. (2015) 133, no. 4, 483–484, 10.1001/jamaophthalmol.2014.5379, 2-s2.0-84928252911, 25569602. 25569602

[11] Khan J. C. and Shuttleworth G. N. , Annular Granular Corneal Opacity: a Rare Corneal Stromal Dystrophy or Degeneration?, British Journal of Ophthalmology. (2000) 84, no. 10, 1205–1206, 10.1136/bjo.84.10.1203c, 11202916. 11202916 PMC1723244

[12] Nguyen D. Q. , Quah S. A. , Kumar N. , Jacob A. , and Kaye S. B. , In-Vivo Scanning of Ascher Intrastromal Corneal Ring Opacity, British Journal of Ophthalmology. (2007) 91, no. 12, 1710–1711, 10.1136/bjo.2006.109322, 2-s2.0-36749085229, 18024816. 18024816 PMC2095549

[13] Almeida R. , Ruão M. , Almeida I. , Rodrigues F. D. , Costa-Ferreira C. , and Chibante-Pedro J. , Bilateral Ring-Shaped Corneal Opacity: Case Report and Review of the Literature, Pan-American Journal of Ophthalmology. (2015) 15, no. 2, 61–62, 10.15234/vpa.v15i2.308.

[14] Rieger G. , Primary unilateral annular opacity of the cornea, Fortschritte der Ophthalmologie: Zeitschrift der Deutschen Ophthalmologischen Gesellschaft. (1987) 84, no. 3, 242–244, 3497857. 3497857

[15] Rohrbach J. M. , Kleiser N. , Kaufmann-Fechner J. , and Lisch W. , Corneal Ring Opacity (Ascher ring)--A Case Report, Klinische Monatsblatter fur Augenheilkunde. (2001) 218, no. 4, 276–278, 10.1055/s-2001-14926, 2-s2.0-0343774057, 11392275. 11392275

[16] Graham B. , Bilateral Idiopathic Corneal Ring Opacity (Ascher Ring), Journal of Medical Optometry. (2024) 2, no. 2, 10.62055/rmykuzqexpfw.

[17] McAlinden C. and Williams C. P. R. , A 54-Year-Old Man With Bilateral Symmetrical Circular Corneal Opacities, Digital Journal of Ophthalmology: DJO. (2021) 26, no. 2, 21–26, 10.5693/djo.03.2019.12.001, 33867878. 33867878 PMC8031963

