# Supplementary figures and images for: Cataract Surgery in a Case of Bilateral Idiopathic Corneal Ascher Rings Associated With Anterior Segment Changes
Source: Case Rep Ophthalmol Med. 2026 Apr 2;2026:8164915. doi: 10.1155/crop/8164915 (PMC13051880; doi:10.1155/crop/8164915)

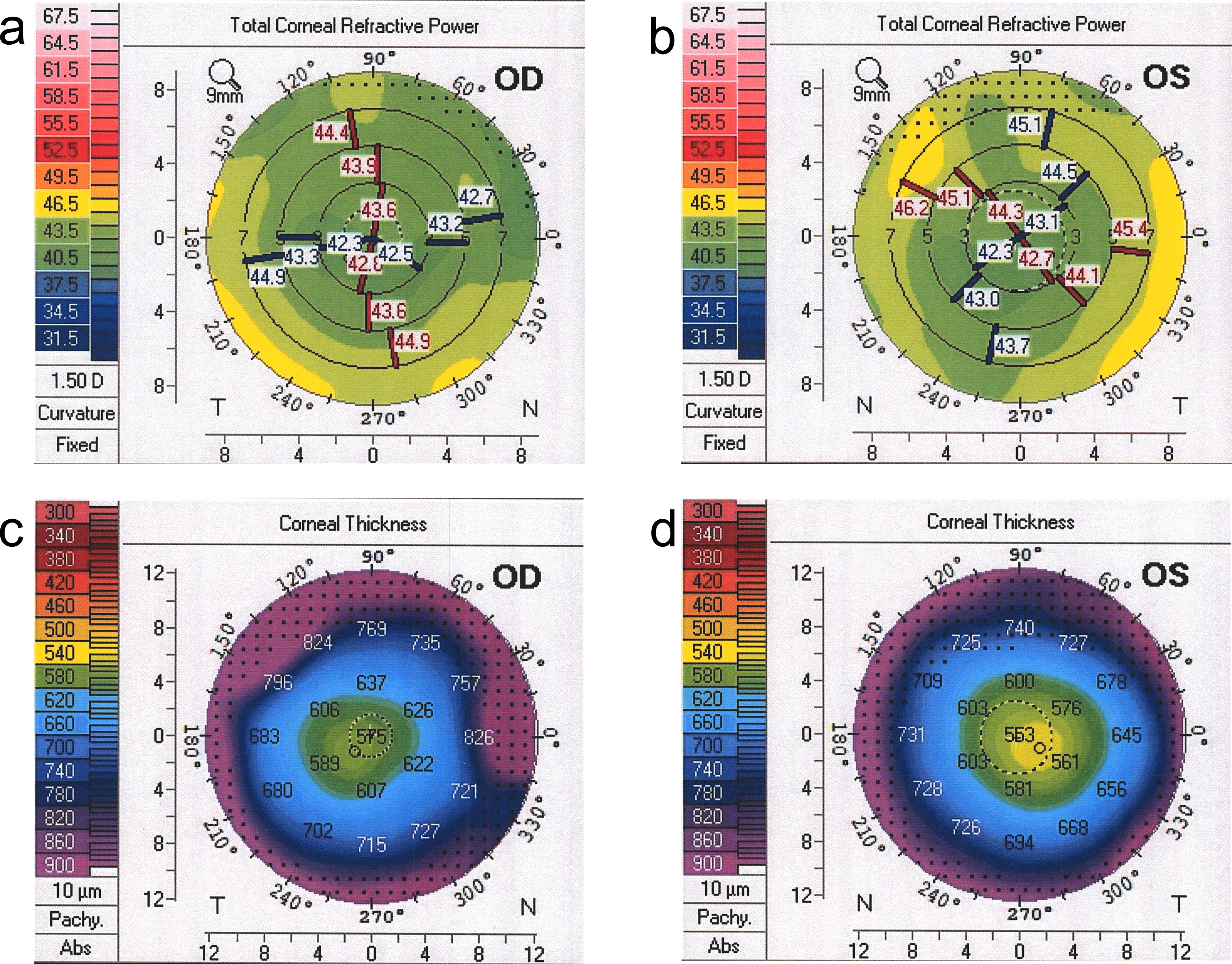

Supplement: Supplementary file 1 — Supporting Information 1 Figure S1: Corneal topography, keratometry and pachymetry data by Pentacam imaging — normal values in both eyes. [file CROP-2026-8164915-s001.jpg]

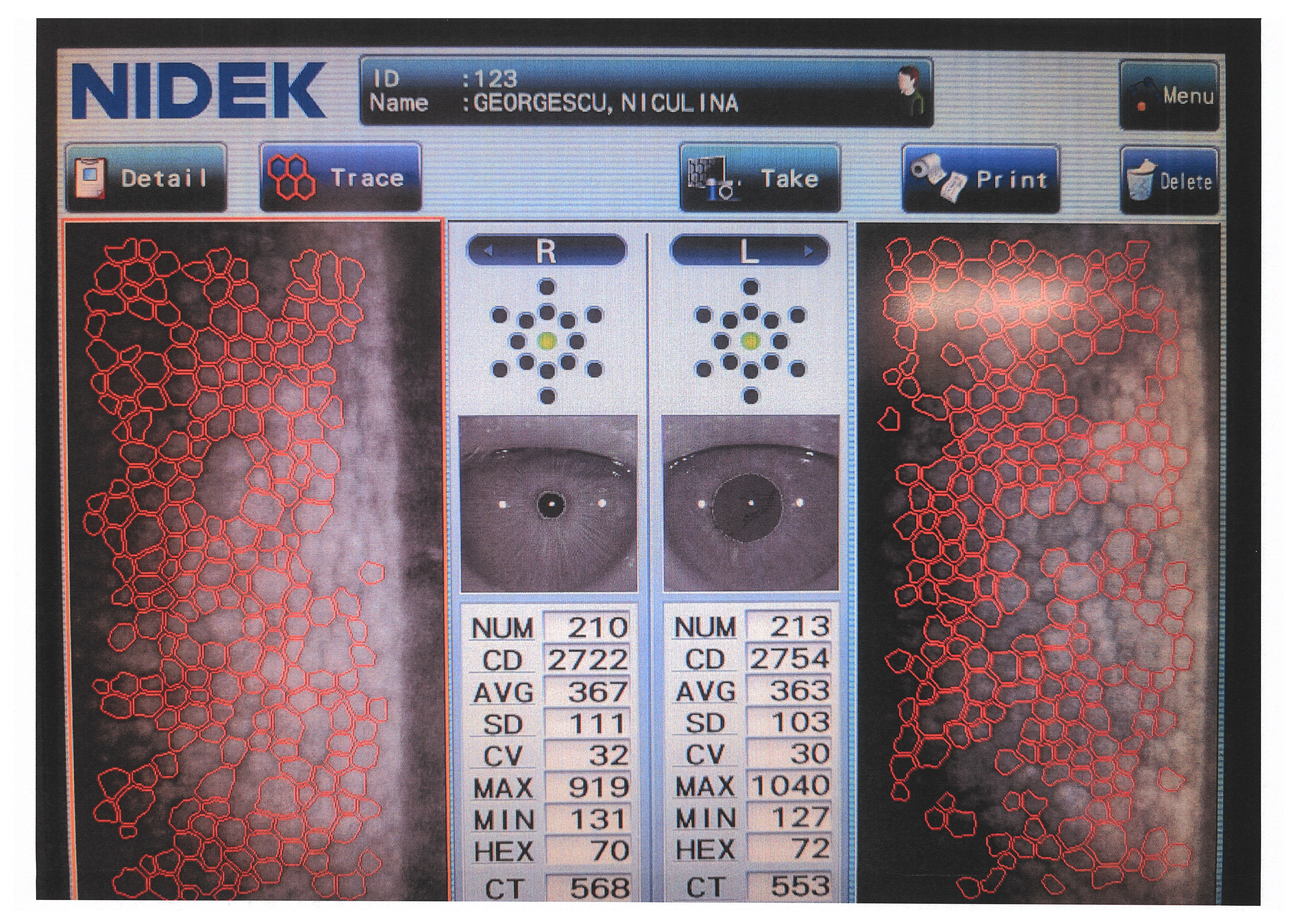

Supplement: Supplementary file 2 — Supporting Information 2 Figure S2: Specular microscopy data (Nidek, CM 530®)— normal range in both eyes. [file CROP-2026-8164915-s002.jpeg]
